# Astrocytes Enhance *Streptococcus suis*-Glial Cell Interaction in Primary Astrocyte-Microglial Cell Co-Cultures

**DOI:** 10.3390/pathogens5020043

**Published:** 2016-06-13

**Authors:** Jana Seele, Roland Nau, Chittappen K. Prajeeth, Martin Stangel, Peter Valentin-Weigand, Maren Seitz

**Affiliations:** 1 Center for Infection Medicine, Institute for Microbiology, University of Veterinary Medicine Hannover, Bischofsholer Damm 15, Hannover 30173, Germany; jana_seele@gmx.de (J.S.); peter.valentin@tiho-hannover.de (P.V.-W.); 2 Institute for Neuropathology, University Medical Center Göttingen, Robert-Koch-Straße 40, Göttingen 37099, Germany; rnau@gwdg.de; 3 Department of Geriatrics, Evangelisches Krankenhaus Göttingen-Weende, An der Lutter 24, Göttingen 37075, Germany; 4 Department of Neurology, Center for Systems Neuroscience (ZSN), Hannover Medical School, Carl-Neuberg-Straße 1, Hannover 30625, Germany; ChittappenKandiyil.Prajeeth@mh-hannover.de (C.K.P.); Stangel.Martin@mh-hannover.de (M.S.)

**Keywords:** astrocytes, microglial cells, co-cultures, bacteria-cell-association, NO release, *S. suis*

## Abstract

*Streptococcus* (*S*.) *suis* infections are the most common cause of meningitis in pigs. Moreover, *S. suis* is a zoonotic pathogen, which can lead to meningitis in humans, mainly in adults. We assume that glial cells may play a crucial role in host-pathogen interactions during *S. suis* infection of the central nervous system. Glial cells are considered to possess important functions during inflammation and injury of the brain in bacterial meningitis. In the present study, we established primary astrocyte-microglial cell co-cultures to investigate interactions of *S. suis* with glial cells. For this purpose, microglial cells and astrocytes were isolated from new-born mouse brains and characterized by flow cytometry, followed by the establishment of astrocyte and microglial cell mono-cultures as well as astrocyte-microglial cell co-cultures. In addition, we prepared microglial cell mono-cultures co-incubated with uninfected astrocyte mono-culture supernatants and astrocyte mono-cultures co-incubated with uninfected microglial cell mono-culture supernatants. After infection of the different cell cultures with *S. suis*, bacteria-cell association was mainly observed with microglial cells and most prominently with a non-encapsulated mutant of *S. suis*. A time-dependent induction of NO release was found only in the co-cultures and after co-incubation of microglial cells with uninfected supernatants of astrocyte mono-cultures mainly after infection with the capsular mutant. Only moderate cytotoxic effects were found in co-cultured glial cells after infection with *S. suis*. Taken together, astrocytes and astrocyte supernatants increased interaction of microglial cells with *S. suis*. Astrocyte-microglial cell co-cultures are suitable to study *S. suis* infections and bacteria-cell association as well as NO release by microglial cells was enhanced in the presence of astrocytes.

## 1. Introduction

*Streptococcus* (*S*.) *suis* is one of the most important porcine pathogens causing meningitis, arthritis, endocarditis, in some cases encephalitis and other pathologies [1,2]. Moreover, it is a zoonotic pathogen. Most human infections occur in Southeast Asia with meningitis as the main pathology [3].

*S. suis* possesses a variety of virulence and virulence-associated factors including the capsule (CPS) and suilysin [4]. The capsule was shown to protect *S. suis* against killing by phagocytes and deposition of complement [5,6,7,8]. Moreover, in pig infection experiments capsular mutants of *S. suis* were completely avirulent [6]. Suilysin, the hemolysin of *S. suis*, is a pore-forming cholesterol-dependent cytolysin and might support *S. suis* to cross epi- and endothelial barriers [9,10].

To cause meningitis *S. suis* has to enter the central nervous system (CNS) via the blood brain barrier (BBB) or the blood cerebrospinal fluid barrier (BCSFB) [9]. Adhesion to and invasion of brain microvascular endothelial cells (part of the BBB) and cells of the plexus chorioideus (part of BCSFB) by *S. suis* were shown *in vitro* [11,12,13,14,15]. Astrocytes form together with endothelial cells the BBB and separate the neuronal parenchyma from non-neuronal cells along the blood vessels and the meninges [16]. Besides providing structural support and nutrients for neuronal cells, [17] astrocytes have barrier functions, liming the spread of infections to the CNS parenchyma, and have pro- as well as anti-inflammatory properties [16]. Although it is hypothesized that astrocytes play a crucial role in host-pathogen interaction during streptococcal meningitis, interactions of streptococci and astrocytes are only poorly investigated [18]. A further glial cell subtype, the microglial cells, represents macrophages of the CNS, which play an important role as phagocytic and antigen-presenting cells [19]. It has been described that activation of microglial cells is modulated by astrocytes [20] and astrocytes are necessary for activation of microglial cells in co-culture e.g., during borna virus infection [21]. Moreover, both cell types respond to bacterial infections of the CNS [22,23,24], have direct contact in brain tissue, and were shown to interact through signaling in cell culture [25,26].

Interaction of *S. suis* with human astrocyte and microglial cell lines as well as with primary murine astrocytes has been previously reported, and an involvement of these cell types in *S. suis* infections of the CNS was shown [27,28,29,30], but so far primary astrocyte and microglial cell co-cultures were not studied. Co-cultures enable analysis of *S. suis* interactions with and between those most abundant and important cell types of the CNS. A further advantage of a murine primary co-culture system is the use of cells from genetically modified animals. For that reason the aim of this study was to establish murine primary astrocyte microglial cell co-cultures for *S. suis* infections and to compare interaction of *S. suis* with mono- and co-cultured astrocytes and microglial cells.

## 2. Results and Discussion

### 2.1. Association of S. suis with Primary Astrocytes and Microglial Cells

**Figure 1:**
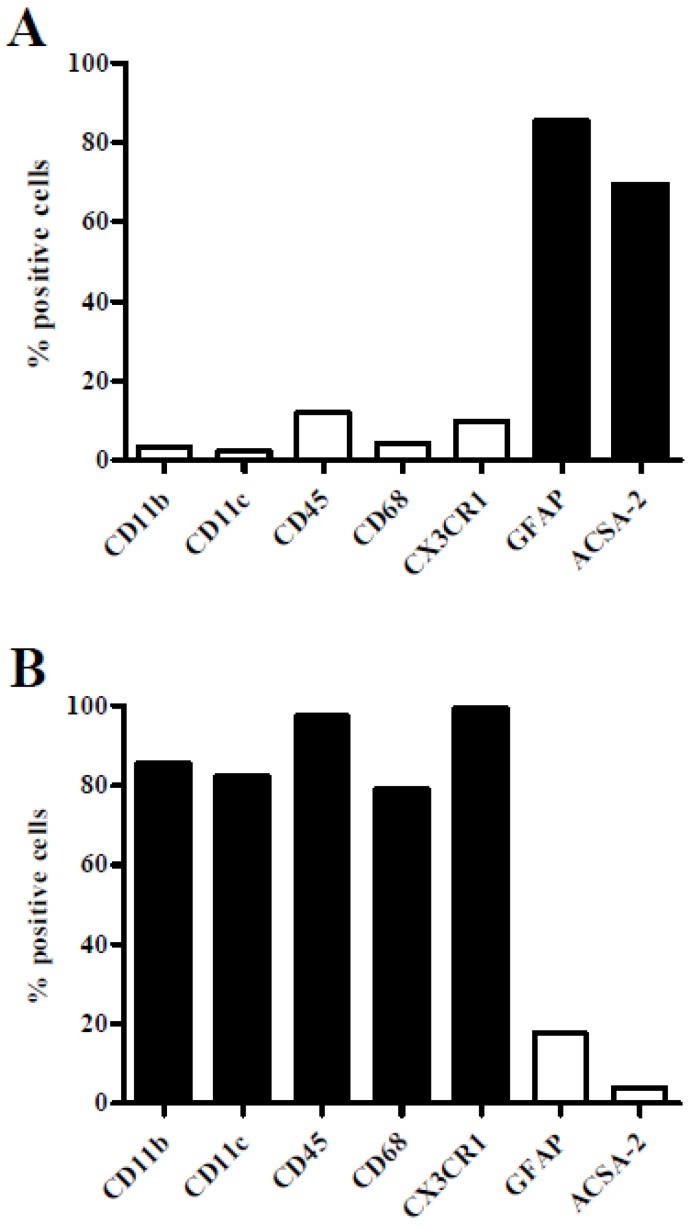
Phenotypical characterisation of primary mouse astrocytes and microglial cells. Flow cytometry analysis of primary astrocytes (**A**) and microglial cells (**B**) isolated from brain tissue of new-born C57BL/6 mice. Cells were stained for cell-surface antigens (CD11b, CD11c, CD45, CX3CR1, and ACSA-2) and intracellular antigens (CD68 and GFAP) as indicated. Means of two independent preparations are shown.

For analysis of *S. suis*-glial cell interactions, primary astrocytes and microglial cells from brain tissue of new-born C57BL/6 mice were characterized by flow cytometry analysis. As shown in Figure 1A astrocytes were positive for the astrocyte cell-surface marker ACSA-2 as well as for the main intermediate filament protein of mature astrocytes (glial fibrillary acidic protein, GFAP). Other classical cell-surface marker proteins (CD11b, CD11c, and CD45) and the cytosolic protein CD68 of immune cells, including microglial cells, were rarely detectable. In contrast, microglial cells (Figure 1B) expressed all common immune cell markers and the transmembrane chemokine receptor CX3CR1, which was absent in astrocytes. Both, GFAP and ACSA-2-positive cells were found in low amounts in microglial cell preparations. Glial cell preparations showing less than 20% contamination of the respective other glial cell type were defined as pure.

**Figure 2:**
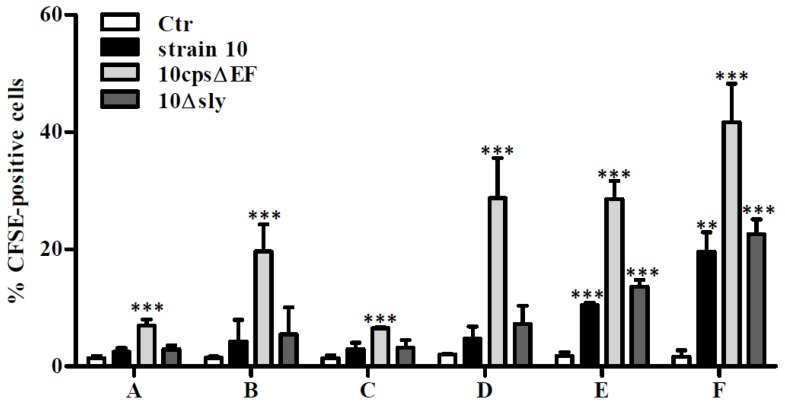
Association of *S. suis* with primary mouse glial cells. Various glial cell culture systems: (**A**) astrocyte mono-culture, (**B**) microglial cell mono-culture, (**C**) astrocyte mono-culture pre-incubated with supernatants (SN) of uninfected microglial cell cultures, (**D**) microglial cell mono-culture pre-incubated with SN of uninfected astrocyte cultures, (**E**) astrocyte-microglial cell co-culture (low amount of microglial cells), and (**F**) astrocyte-microglial cell co-culture (high amount of microglial cells), respectively, were infected with CFSE-labeled *S. suis* strain 10, 10*cps*Δ*EF*, or 10Δ*sly* at a MOI of 10:1. Percentage of CFSE-positive cells were measured by flow cytometry. Results are expressed as means with standard deviation (SD) of three independent experiments, and statistically significant differences when compared to uninfected control cells are indicated by ** (*p*-value < 0.01), and *** (*p*-value < 0.001), one-way analysis of variance (ANOVA) followed by a Dunnett post-hoc test.

To further study *S. suis*-glial cell interactions, pure astrocyte and microglial cell preparations were used in various cell culture systems. At first, astrocytes and microglial cells were maintained separately in mono-cultures. In addition, astrocyte mono-cultures were pre-incubated with supernatants of uninfected microglial cell cultures and microglial mono-cultures were pre-stimulated with supernatants of uninfected astrocyte mono-cultures, respectively. At last, astrocytes and microglial cells were combined in co-cultures using two different amounts of microglial cells. After one day of cultivation co-cultures reached a final microglial cell:astrocyte ratio of 1:2 (low amount of microglial cells) and 1:1 (high amount of microglial cells), respectively. These various glial cell cultures were infected with carboxyfluorescein succinimidyl ester (CFSE)-stained *S. suis* serotype 2 wildtype (wt) strain 10, its non-encapsulated mutant strain 10*cps*Δ*EF* and a suilysin-deficient strain 10Δ*sly*, respectively, at a multiplicity of infection (MOI) of 10:1 for 2 h. Afterwards glial cells were harvested and CFSE-positive cells were quantified by flow cytometry. In all mono-cultured glial cell cultures, irrespectively of the pretreatment with uninfected glial cell supernatants, only the non-encapsulated strain showed a significant bacteria-cell association (Figure 2A–D). Higher association rates were found in microglial cell mono-cultures (Figure 2B; 19.6%) than in astrocyte mono-cultures (Figure 2A; 6.9%). Nevertheless, pretreatment of microglial cell mono-cultures with supernatants of uninfected astrocyte mono-cultures increased the association rate of 10*cps*Δ*EF* to 28.7% (Figure 2D). A comparable number of CFSE-positive cells (Figure 2E; 28.6%) was found in the 10*cps*Δ*EF*-infected astrocytes-microglial co-culture with low amount of microglial and the highest bacteria-cell association for 10*cps*Δ*EF* was observed in the co-culture with a high amount of microglial cells (Figure 2F; 41.6%). In contrast, both encapsulated *S. suis* strains (strain 10 and 10Δ*sly*) showed only in the co-cultures a lower but significant and comparable glialcell association (Figure 2E,F). To further elucidate the preferred cell type for interaction, *S. suis*-infected glial cells were additionally stained after harvesting with the cell-surface markers ACSA-2 and CX3CR1 and analyzed by flow cytometry.

**Figure 3:**
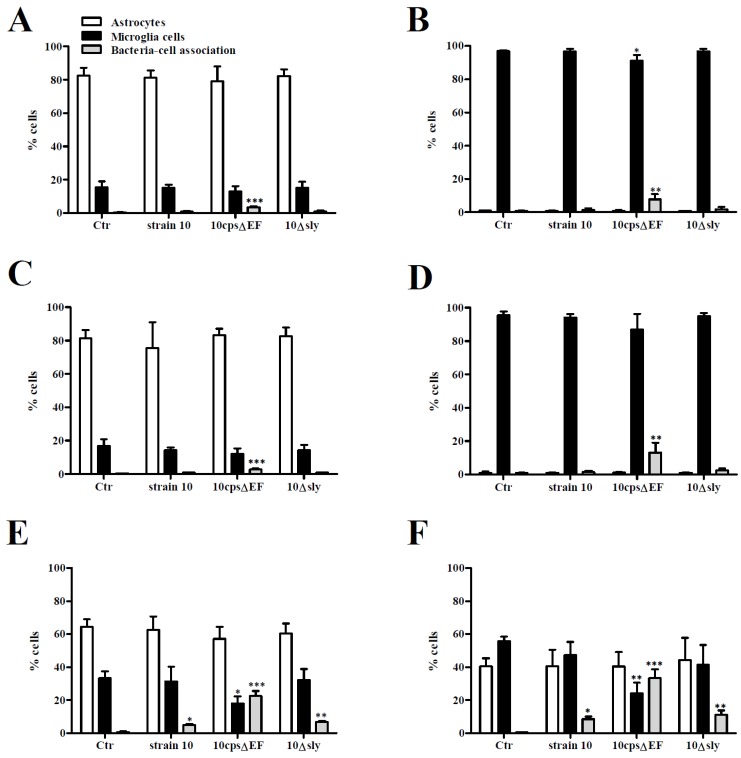
Association of *S. suis* with primary mouse astrocytes and microglial cells. Various glial cell culture systems: (**A**) astrocyte mono-culture; (**B**) microglial cell mono-culture; (**C**) astrocyte mono-culture pre-stimulated with SN of uninfected microglial cell cultures; (**D**) microglial cell mono-culture pre-stimulated with SN of uninfected astrocyte cultures; (**E**) astrocyte-microglial cell co-culture (low amount of microglial cells); and (**F**) astrocyte-microglial cell co-culture (high amount of microglial cells), were infected with CFSE-labeled *S. suis* strain 10, 10*cps*Δ*EF*, or 10Δ*sly* at a MOI 10:1 for 2 h. Astrocytes and microglial cells were stained for the cell-surface antigens ACSA-2 and CX3CR1, respectively. Relative amounts (%) of astrocytes (white bars), microglial cells (black bars) and microglial cells/astrocytes in association with bacteria (CFSE-positive, grey bars) were determined by flow cytometry analysis. Results are expressed as means with SD of three independent experiments, and statistically significant differences when compared to uninfected control cells are indicated by * (*p*-value < 0.05), ** (*p*-value < 0.01), and *** (*p*-value < 0.001), one-way-ANOVA followed by a Dunnett post-hoc test.

To distinguish between astrocytes and microglial cells, analyzed cells were divided into three groups according to their specific staining profile: (i) astrocytes (ACSA-2-positive); (ii) microglial cells (CX3CR1-positve); and (iii) glial cells in association with bacteria (ACSA-2 or CX3CR1-positive plus CFSE-positive). In good agreement with the results shown in Figure 2, in glialcell mono-cultures only the non-encapsulated strain interacted with glial cells (Figure 3A–D). In 10*cps*Δ*EF*-infected microglial cell mono-cultures (with and without pre-stimulation with supernatants of uninfected astrocyte mono-cultures) the CFSE-positive cell fraction was associated with a decreased number of microglial cells (Figure 3B,D). Thus, the non-encapsulated mutant most likely interacted with microglial cells. As described above, in case of a 10*cps*Δ*EF* infection, pretreatment of microglial cell mono-cultures with supernatants of uninfected astrocyte mono-cultures further increased association of glial cells and bacteria (Figure 3B; 3.9% and 3D; 13.1%), whereas pretreatment of astrocyte mono-cultures with supernatants of uninfected microglial cell mono-cultures did not influence bacteria-cell association rates of 10*cps*Δ*EF* (Figure 3A; 3.3% and 3C; 2.7%).

Astrocytes are known to produce factors interacting with microglial cells. Min *et al.* [20] detected heat labile compounds in astrocyte supernatants, smaller than 3 kDa, influencing microglial cells in case of preventing excessive brain inflammation, whereas in an astrocyte-microglial cell co-cultures studying borna virus infection heat resistant factors activated microglial cells [21]. Moreover, astrocytes secret a variety of proteins like trophic factors, cytokines, chemokines, proteases, and protease inhibitors [31].

One could asked, that supernatants of uninfected glial cells may not contain certain mediators/components to subsequently efficiently activate the other glial cell type, thus pre-stimulation of glial cell mono-cultures was also performed with supernatants of astrocytes or microglial cells 24 h pre-infected with *S. suis* 10*cps*Δ*EF*. Surprisingly, no differences in bacteria-cell association were observed between uninfected and pre-infected glial cell supernatants (data not shown). Thus, bacterial pre-infection of astrocytes and microglial cells seems to play a minor role in astrocytes-microglialcell interaction.

In contrast to 10*cps*Δ*EF*, after infection with strain 10 or 10Δ*sly* almost no CFSE-positive cell fraction was found in any glial cell mono-culture (with and without pre-incubation with supernatants of uninfected glial cell mono-cultures) (Figure 3A–D). Bacterial replication during infection was monitored and confirmed that the observed effects were not due to differences in growth properties of the different strains (Figure S1).

As expected, the amount of glial cells recovered from the uninfected astrocyte-microglial cell co-cultures reflected the initial seeding proportions (Figure 3E; microglial cells: astrocytes 1:2 and 3F; 1:1). In these co-cultures most CFSE-positive cells were observed after infection with 10*cps*Δ*EF*, and the fraction of glial cells in association with bacteria increased with the amount of microglial cells added to the co-culture (Figure 3E; 22.6% and 3F; 33.3%). Of importance, the amount of astrocytes remained constant, but the percentage of microglial cells significantly decreased in parallel with an increasing CFSE-positive cell fraction after infection with 10*cps*Δ*EF*. Thus, microglial cells were the predominant interaction partner for *S. suis*. Contrary to mono-cultures, in co-cultures both encapsulated *S. suis* strains were found in association with glial cells, but to a lower extent. The role of suilysin in bacteria-cell interaction is still controversially discussed and highly dependent on the cell type [13,32,33,34,35,36]. Similar to a previous study in a murine microglial cell line [27], but unlike with human astrocytes [30], suilysin seemed to play a minor role in bacteria-cell association, since differences between strain 10 (Figure 3E; 5.0% and 3F; 8.6%) and the suilysin-deficient mutant were marginal (Figure 3E; 6.8% and 3F; 11.1%). Since microglial cells are professional phagocytes of the brain, most likely *S. suis* is phagocytosed by these cells rather than only be attached to them. Hence, a higher association rate of 10*cps*Δ*EF* with microglial cells in comparison to the tested encapsulated strains is plausible, since the protecting properties of the capsule against phagocytosis are missing. Furthermore, it has been described that primary murine astrocytes are unable to phagocytose even a non-encapsulated strain of *S. suis* [28]. Taken together, *S. suis* interacts mainly with microglial cells and *S. suis*-microglial cell association was facilitated by the presence of astrocytes. Furthermore, association was negatively affected by the capsule, but independent of the production of suilysin.

### 2.2. NO Release by Primary Mouse Glial Cells after Infection with S. suis

**Figure 4:**
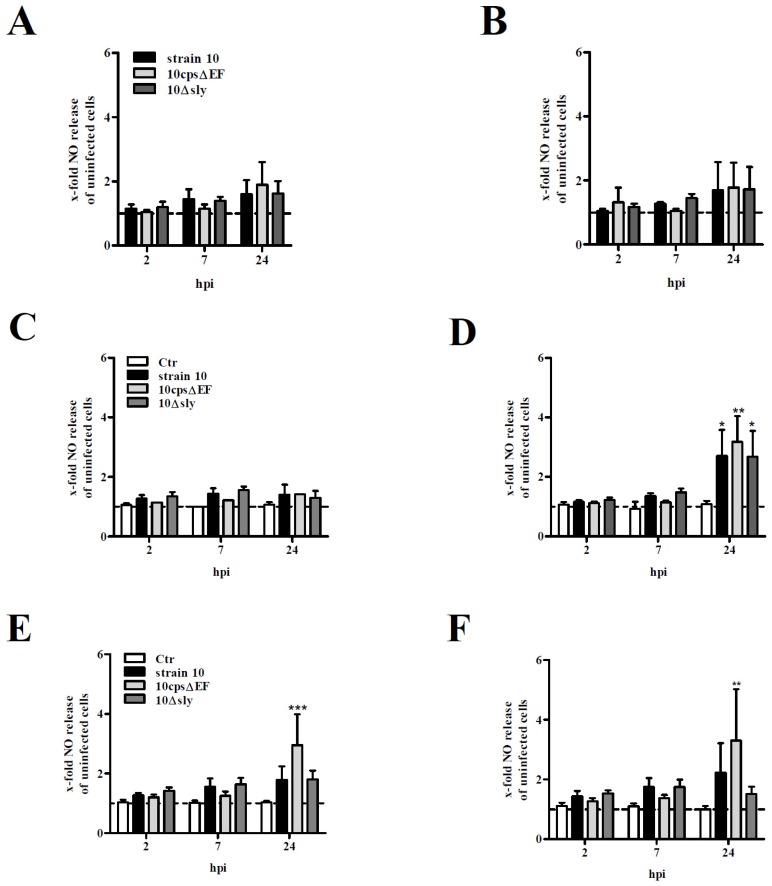
NO release by primary mouse glial cell cultures after infection with *S. suis*. Various glial cell culture systems: (**A**) astrocyte mono-culture; (**B**) microglial cell mono-culture; (**C**) astrocyte mono-culture pre-stimulated with SN of uninfected microglial cell cultures; (**D**) microglial cell mono-culture pre-stimulated with SN of uninfected astrocyte cultures; (**E**) astrocyte-microglial cell co-culture (low amount of microglial cells); and (**F**) astrocyte-microglial cell co-culture (high amount of microglial cells), were infected with *S. suis* strain 10, 10*cps*Δ*EF*, or 10Δ*sly* at a MOI of 10:1 for 2, 7, and 24 h (hpi). Nitirc oxide (NO) release was measured using Griess reagent. All results are expressed as x-fold NO release normalized to uninfected astrocytes or microglial cells which were not pre-incubated with SN (dashed line). In the co-culture system values were normalized to uninfected astrocytes. Uninfected astrocytes or microglial cells pre-incubated with SN only or co-cultured served as further controls (Ctr, **C**–**F**). Results are expressed as means with SD of four (**A** + **B**) or six (**C**–**F**) independent experiments and statistically significant differences when compared to uninfected astrocytes and microglial cells (**A**,**B**), uninfected astrocytes and microglial cells pre-incubated with supernatants (**C**,**D**) or astrocyte-microglial cell co-cultures (**E**,**F**) are indicated by * (*p*-value < 0.05), ** (*p*-value < 0.01), and *** (*p*-value < 0.001), one-way-ANOVA followed by Dunnett post-hoc test.

Reactive glial cells can produce a variety of second mediators, such as cytokines, neurotransmitters or nitic oxide (NO) that are involved in neuroinflammatory and -degenerative processes [37]. To analyze NO release by astrocytes and microglial cells as well as to study the possible interaction of both glial cell types, the glial cell cultures introduced above were infected at a MOI of 10:1 for 2, 7 and 24 h. At early time points post infection (2 and 7 h post infection (hpi)) no significant NO levels were detectable in any glial cell culture (Figure 4). As shown in Figure 4A,B even after 24 hpi no significant NO release was observed by mono-cultured astrocytes or microglial cells infected with *S. suis*. Interestingly, also pre-stimulation of astrocytes with supernatants of uninfected microglial cell mono-cultures failed to induce NO release (Figure 4C), but an efficient NO release was measured 24 hpi in microglial cell cultures pre-incubated with supernatants of uninfected astrocyte mono-cultures (Figure 4D). The capsular mutant 10*cps*Δ*EF* induced the highest NO levels when compared to uninfected microglial cells pre-incubated with the supernatant of uninfected astrocytes (control, Ctr) (3.2-fold). Both encapsulated *S. suis* strains 10 and 10Δ*sly* showed a 2.7-fold higher NO release. To test the direct effect of astrocytes on the NO release by microglial cells, both co-culture systems were used. 24 hpi significantly higher NO levels were measured in co-cultures infected with *S. suis* strain 10*cps*Δ*EF* (Figure 4E,F). In accordance to the results described above, the level of released NO in the co-cultures was dependent on the number of microglial cells added to the system. Strain 10*cps*Δ*EF* induced a 3.0-fold (low amount of microglial cells) and 3.3-fold (high amount of microglial cells) NO release, respectively, when compared to uninfected co-cultures. Thus, NO seems to be mainly produced by microglial cells, but astrocytes are indirectly (soluble factors in the supernatant) or directly (cellular contact) crucial for a sufficient NO release by microglial cells after infection with *S. suis*. In fact, microglial cells are accepted as the main glial cell type responsible for NO release, e.g., upon lipopolysaccharide (LPS)stimulation or *S. suis* infection [37]. Nevertheless, astrocytes appear to be capable of expressing inducible nitric oxide synthase (iNOS) [28,37]. In addition, in a previous study, NO release by LPS-stimulated microglial cells was described to be enhanced in the presence of astrocytes [37].

In contrast to 10*cps*Δ*EF*, the increase of the NO release caused by the encapsulated *S. suis* strains in the co-cultures, was small and failed to reach statistical significance (Figure 4E,F). Differences observed between encapsulated and non-encapsulated *S. suis* strains in the ability to induce NO were not related to differences in growth, since replication rates of all tested strains were similar at all tested time points (Figure S1). Nevertheless, as described above encapsulated *S. suis* strains were able to induce a NO release by microglial cells when pre-incubated with supernatants of uninfected astrocyte mono-cultures (Figure 4D). This might be due to the fact that supernatants of astrocyte mono-cultures for pre-incubation were harvested after 3–5 days after cultivation whereas co-cultures were only incubated for 1 day. Thus, soluble factors stimulating NO release by microglial cells might be higher concentrated in supernatants of astrocyte mono-cultures than in the co-cultures.

The masking effect of the capsule, which might hide cell surface structures crucial for microglial cell activation, may explain why the non-encapsulated mutant of *S. suis* induced the highest NO levels [27,28]. For instance, cellwall components such as lipoteichoic acid (LTA) of Gram-positive bacteria are known to activate NO in microglial cells [38]. Nevertheless, knowledge of receptors and mechanisms involved in the activation of microglial cells infected with *S. suis* is limited. First results indicate that Toll-like receptor 2 (TLR-2), nucleotide oligomerization domain 2 (NOD)-2 and signal transducer and activator of transcription 3 (STAT3) signaling are involved in interaction with *S. suis* as well as mitogen-activated protein kinases (MAPK) signaling events and nuclear factor kappa-light-chain-enhancer of activated B cells (NF-κB) activation [27,29]. Since we found a higher association rate as well as higher levels of NO in microglial cells after infection with the non-encapsulated *S. suis* strain, one could speculate that phagocytosis of *S. suis* might be necessary to subsequent induce sufficient NO release by microglial cells. In our glial cell cultures astrocytes showed an important supportive function to stimulate phagocytosis and NO release by microglial cells. Thus, both glial cell types seem to interact with each other during infection with *S. suis*.

**Figure 5:**
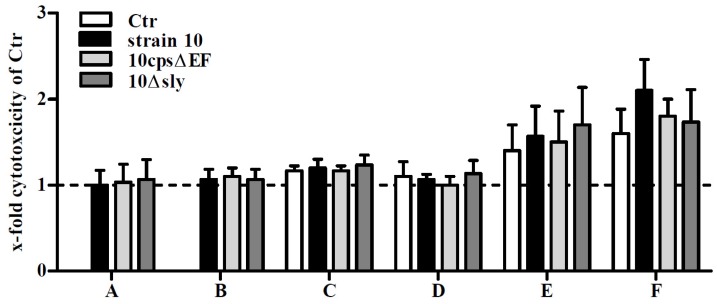
Cell viability of primary mouse glial cell cultures after infection with *S. suis*. Various glial cell culture systems: (**A**) astrocyte mono-culture; (**B**) microglial cell mono-culture; (**C**) astrocyte mono-culture pre-stimulated with SN of uninfected microglial cell cultures; (**D**) microglial cell mono-culture pre-stimulated with SN of uninfected astrocyte cultures; (**E**) astrocyte-microglial cell co-culture (low amount of microglial cells); and (**F**) astrocyte-microglial cell co-culture (high amount of microglial cells), were infected with *S. suis* strain 10, 10*cps*Δ*EF*, or 10Δ*sly* at a MOI 10:1. 24 h post infection cell viability was determined by standard lactate dehydrogenase (LDH) release assay. Results are expressed as x-fold cytotoxicity normalized to uninfected astrocytes and microglial cells which were not pre-incubated with SN (dashed line). In the co-culture system values were normalized to uninfected astrocytes. Uninfected astrocytes or microglial cells pre-stimulated with SN only or co-cultured served as further controls (Ctr, **C**–**F**). Results are expressed as means with SD of three independent experiments. No significant differences when compared to uninfected astrocytes and microglial cells (**A**,**B**), uninfected astrocytes and microglial cells pre-incubated with supernatants (**C**,**D**) or astrocyte-microglial cell co-cultures (E,F) were found, one-way-ANOVA followed by a Dunnett post-hoc test.

NO can have either beneficial (cytoprotective) or detrimental (cytotoxic) effects [39]. To test cytotoxic effects by the infection, which may depend on the NO release, a classical LDH release test was performed 24 hpi (Figure 5). Only moderate cytotoxic effects were observed in both co-culture systems without infection reflecting viable astrocytes and microglial cells and after infection with *S. suis*. In good agreement, cytotoxic effects lower than 1.5% were found in primary astrocyte cultures 24 h after infection with *S. suis* [28]. In contrast, *S. suis* infection led to a time-dependent increase in cytotoxicity levels in BV2 microglial cell line [29]. Interestingly, cytotoxicity was independent of the presence of suilysin, because the suilysin-positive strain 10 and its suilysin-deficient mutant behaved the same way. Nevertheless, since no LDH release was found in *S. suis*-infected microglial cell mono-cultures pre-stimulated with supernatants of uninfected astrocytes, which showed a significant NO release after infection, no correlation between cytotoxic effects and NO could be found.

## 3. Experimental Section

### 3.1. Bacterial Strains and Growth Conditions

*S. suis* strain 10 is a virulent serotype 2 strain (sequence type 1) that has previously been used for experimental infections of piglets and for generation of isogenic mutants [6,40,41,42]. It expresses the virulence-associated muramidase-released protein (MRP), the extracellular factor (EF) and suilysin [43]. The capsule-deficient isogenic mutant 10*cps*∆*EF* is attenuated in virulence. It was originally generated through insertion of a spectinomycin-resistance cassette in the genes *cps*2E and *cps*2F [6]. The suilysin-deficient mutant, strain 10Δ*sly*, was constructed by insertion of an erythromycin cassette in the *sly* gene of strain 10 using the plasmid pBlue/sly/erm as previously described [33]. Streptococci were grown on Columbia blood agar plates for 16 h or in Bacto^TM^ Todd Hewitt broth (THB) to logarithmic growth phase (optical density at 600 nm of 0.6) at 37 °C under aerobic conditions.

### 3.2. Isolation and Cultivation of Mixed Glial Cell Cultures

Mice were cared for in accordance with the principles outlined in the European Convention for the Protection of Vertebrate Animals Used for Experimental and Other Scientific Purposes (European Treaty Series, no. 123 [44]). Mixed glial cells were isolated from brains of newborn (0–2 days postnatal) wild-type C57BL/6 mice and cultivated as previously described [45]. Briefly, meninges of murine brains were removed, whole brains were treated with 0.25% trypsine (Biochrom, Berlin, Germany; 2.5% solution) in HBSS (Sigma-Aldrich, Taufkirchen, Germany) for 10 min at 37 °C, DNAse (Sigma-Aldrich, Taufkirchen, Germany) was added in a concentration of 0.4 mg/mL, and brains were minced by pipetting up and down with a 10 mL pipette. After centrifugation (750× *g*, 8 min) and removal of supernatant cells of two brains were seeded into a 75 cm^2^ cell culture flask and cultured in DMEM 4.5 g/L glucose (Gibco, Karlsruhe, Germany) supplemented with 10% heat-inactivated FCS, 5 mM glutamine, and 100 U/mL penicillin and 100 μg/mL streptomycin at 37 °C + 5% CO_2_ for 10 days or until confluency of the cell layer was reached.

### 3.3. Cultivation and Separation of Astrocytes and Microglial Cells

After 10 days of mixed glial cell cultivation the medium was changed to antibiotic-free conditions. Supernatants of fibroblast cell line L929 were added to a final volume of 30% [v/v]. After 5 days of stimulation microglial cells were harvested by shaking (150 rpm/min) for 30 min at 37 °C and seeded into 24- or 6-well plates in a concentration of 1 × 10^5^ cells/well or 4 × 10^5^ cells/well for microglial cell mono-cultures, respectively. The remaining glial cell culture was again stimulated with supernatants of fibroblast cell line L929 and harvesting of microglial cells was repeated at most five times. For astrocyte cultures the dividing cells in the mixed glial cell cultures were removed by treatment with 100 µm AraC for 72 h (Sigma, Taufkirchen, Germany) as described elsewhere [46]. Subsequently, astrocytes were harvested by trypsin digestion and seeded into 24- or 6-well plates in a concentration of 1 × 10^5^ cells/well or 4 × 10^5^ cells/well for astrocyte mono-cultures, respectively. For co-cultivation of astrocytes and microglial cells, 1 × 10^5^ or 4 × 10^5^ astrocytes/well were seeded into 24- or 6-well plates, respectively. After 24 h of incubation at 37 °C + 5% CO_2_ 1 or 2 × 10^5^ microglial cells/well (24-well plate; low and high microglial cell number, respectively) and 4 or 8 × 10^5^ microglial cells/well (6-well plate, low or high microglial cell number, respectively) were seeded on top of the astrocytes and incubated for further 24 h at 37 °C + 5% CO_2_.

### 3.4. Pre-incubation of Astrocytes and Microglial Cells with Cell Supernatants

Astrocytes and microglial cells were pre-incubated with supernatants of microglial cell and astrocyte mono-cultures, respectively, to investigate the influence of soluble factors on activity of these two cell types. Supernatants of both cell types were harvested after 3–5 days of cultivation. Cells were pre-incubated with supernatants for 24 h at 37 °C + 5% CO_2_.

### 3.5. Flow Cytometry Characterization of Astrocytes and Microglial Cells

Astrocytes and astrocyte-microglial cell co-cultures were harvested by trypsin treatment and microglial cells by scraping. Approximately 3 × 10^5^ cells of each cell type were stained with antibodies against extracellular markers: anti-CD11b-APC, anti-CD11c-PE, anti-CD45-FITC, anti-ACSA-2-PE (Miltenyi Biotec, Bergisch Gladbach, Germany) and anti-CX3CR1-FITC (BioLegend, Koblenz, Germany) as recommended by the manufacturer. For intracellular staining of CD68 and GFAP cells were treated with the Intracellular Fixation & Permeabilization Buffer Set (Affymetrix eBioscience, Frankfurt am Main, Germany) as recommended by the manufacturer and subsequently stained with anti-CD68-APC and anti-GFAP-PE (Miltenyi Biotec, Bergisch Gladbach, Germany) as recommended by the manufacturer. Cells were measured with a Guava^®^ easyCyte flow cytometer (Merck Millipore, Darmstadt, Germany) and analyzed with the guava InCyte™ Software. For each sample 5000 events were acquired and analyzed in a dot plot or histogram.

### 3.6. Flow Cytometry Analysis of Bacteria-Cell-Association

For this purpose, streptococcal strains were labeled with the carboxyfluorescein succinimidyl ester. (CFSE) using CellTrace™ CFSE Cell Proliferation Kit (Invitrogen, Karlsruhe, Germany). Labeling was performed as described [32].

Mono- or co-cultured cells with or without pre-stimulation with cell supernatants were infected with CFSE-labeled *S. suis* with a multiplicity of infection (MOI) of 10:1 (bacterium:cell) for 2 h. Subsequently, the supernatant was removed and the cells were washed three times with PBS. Astrocytes and astrocyte-microglial cell co-cultures were harvested by trypsin treatment and microglial cells by scraping. Following, astrocytes were stained with anti-ASCA-2-PE (Miltenyi Biotec, Bergisch Gladbach, Germany) and microglial cells with anti-CX3CR1-APC (BioLegend, Koblenz, Germany) as described above. Cells were measured with a Guava^®^ easyCyte flow cytometer (Merck Millipore, Darmstadt, Germany) and analyzed with the guava InCyte™ Software. Furthermore, glial cells in association with bacteria (ACSA-2 or CX3CR1-positive plus CFSE-positive) were determined. For each sample 5000 events were acquired and analyzed in a dot plot or histogram.

### 3.7. NO Measurement

Mono- or co-cultured cells with or without pre-incubation with cell supernatants were infected with *S. suis* with a MOI of 10:1 (bacterium:cell) for 2, 7 or 24 h. Nitrite was measured in cell supernatants using Griess reaction (Thermo Fisher Scientific, Darmstadt, Germany) as recommended by the manufacturer.

### 3.8. Evaluation of Cytotoxic Effects

Cytotoxicity was detected by lactate dehydrogenase (LDH) release assay after 24 h of infection as described previously [47].

### 3.9. Statistical Analysis

If not stated otherwise experiments were performed at least three times and one-way analysis of variance (ANOVA) followed by a Dunnett or Turkey post-hoc test was used. Probabilities lower than 0.05 were considered significant (*p* < 0.05 *, *p* < 0.01 ** and *p* < 0.001 ***).

## 4. Conclusions

In conclusion, *S. suis* interacts poorly with mono-cultured microglial cells and astrocytes, but infection of microglial cells co-cultured with (supernatants of) astrocytes leads to the induction of NO release and *S. suis* is found in association with these cells. Bacteria-cell association as well as NO release by microglial cells is efficiently enhanced in the presence of astrocytes and influenced by the presence of the capsule, since the non-encapsulated mutant shows the highest effects. These results underline the important function of astrocytes during bacterial infection of the CNS and the need to investigate complex *in vitro* models. Further experiments are needed to identify bacterial and host cell factors involved in the crosstalk between microglial cells and astrocytes to analyze the underlying mechanisms of astrocyte-microglialcell interaction in *S. suis* CNS infections.

## Supplementary Materials

The following are available online at www.mdpi.com/2076-0817/5/2/43/s1, Figure S1: Replication rates of *S. suis* strain 10, 10Δ*sly* and 10*cps*Δ*EF* on primary mouse glial cell cultures.
