# Supplementary material for: Astrocytes Enhance Streptococcus suis-Glial Cell Interaction in Primary Astrocyte-Microglial Cell Co-Cultures
Source: Pathogens. 2016 Jun 13;5(2):43. doi: 10.3390/pathogens5020043 (PMC4931394; doi:10.3390/pathogens5020043)
Supplement: Supplementary file 1 [file pathogens-05-00043-s001.pdf]

Supplementary Materials: Astrocytes Enhance Streptococcus Suis-Glia Cell Interaction in Primary Astrocyte-Microglial Cell Co-Cultures

Jana Seele, Roland Nau, Chittappen K. Prajeeth, Martin Stangel, Peter Valentin-Weigand and Maren Seitz

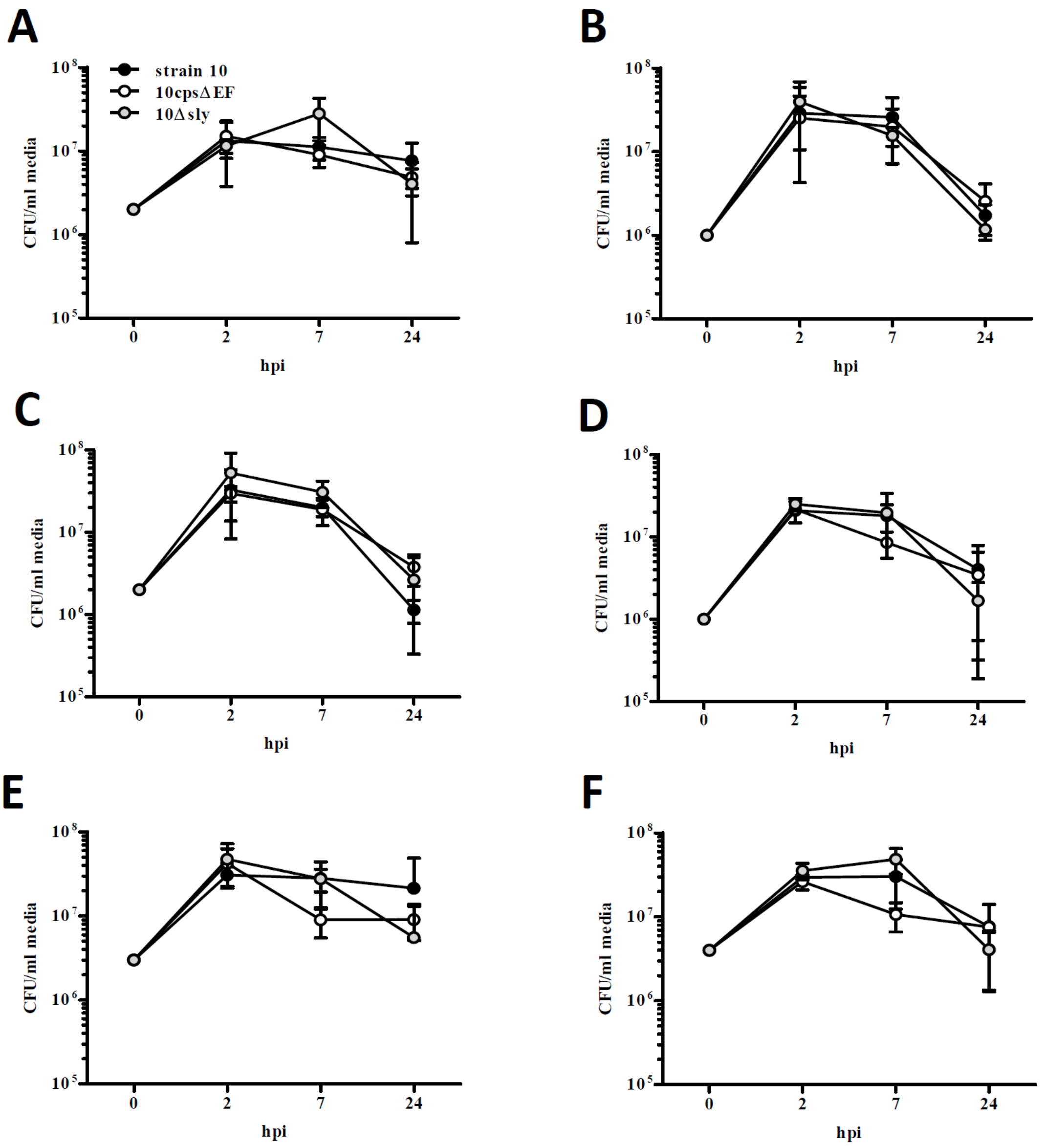

Figure S1. Replication rates of *S. suis* strain 10, 10Δ*sly* and 10*cps*Δ*EF* on primary mouse glia cell cultures. Growth kinetics of streptococci determined by counting of colony forming units (CFU) in the supernatants of infected glia cell culture systems: (**A**) astrocyte mono-culture; (**B**) microglial cell mono-culture; (**C**) astrocyte mono-culture pre-incubated with supernatant (SN) of uninfected microglial cell culture; (**D**) microglial cell mono-culture pre-incubated with SN of uninfected astrocytes cultures; (**E**) astrocyte-microglial cell co-culture (low amount of microglial cells); and (**F**) astrocyte-microglial cell co-culture (high amount of microglial cells), respectively, after replica plating on blood agar plates. Primary mouse glia cells cultures were infected with *S. suis* strain 10, 10*cps*Δ*EF*, or 10Δ*sly* at a MOI of 10:1 and supernatant of infected cells were plated at indicated time points. Growth of *S. suis* was recorded over a period of 24 h. No significant differences were found, one-way-ANOVA followed by a Tukey post-hoc test.
